# Oral Antiviral Treatment for COVID-19: A Comprehensive Review on Nirmatrelvir/Ritonavir

**DOI:** 10.3390/v14112540

**Published:** 2022-11-17

**Authors:** Karolina Akinosoglou, Georgios Schinas, Charalambos Gogos

**Affiliations:** Department of Internal Medicine, Medical School, University of Patras, 26504 Rio, Greece

**Keywords:** COVID-19, nirmatrelvir/ritonavir, Paxlovid, SARS-CoV-2, nirmatrelvir, ritonavir, treatment

## Abstract

Despite the rapid development of efficient and safe vaccines against COVID-19, the need to confine the pandemic and treat infected individuals on an outpatient basis has led to the approval of oral antiviral agents. Taking into account the viral kinetic pattern of SARS-CoV-2, it is of high importance to intervene at the early stages of the disease. A protease inhibitor called nirmatrelvir coupled with ritonavir (NMV/r), which acts as a CYP3A inhibitor, delivered as an oral formulation, has shown much promise in preventing disease progression in high-risk patients with no need for supplemental oxygen administration. Real-world data seem to confirm the drug combination’s efficacy and safety against all viral variants of concern in adult populations. Although, not fully clarified, viral rebound and recurrence of COVID-19 symptoms have been described following treatment; however, more data on potential resistance issues concerning the Mpro gene, which acts as the drug’s therapeutic target, are needed. NMV/r has been a gamechanger in the fight against the pandemic by preventing hospitalizations and halting disease severity; therefore, more research on future development and greater awareness on its use are warranted.

## 1. Introduction

Severe acute respiratory syndrome coronavirus 2, most commonly known as SARS-CoV-2, is an RNA virus first detected in Wuhan, China, in December 2019 that has posed a significant burden on health systems worldwide with its spread, which led to the coronavirus disease 2019 (COVID-19) pandemic. In the following two years, the coronavirus disease resulted in significant mortality and morbidity, especially in people with chronic conditions and multiple comorbidities. Clinical manifestations regarding COVID-19 are mainly driven by the biphasic nature of the disease, including an initial viral replication and toxicity stage, followed by a second phase of inflammatory response [1]. The latter is responsible for the complicated disease, as reflected by severe pneumonia, respiratory failure, the need for hospitalization, mechanical ventilation, and death. 

Following SARS-CoV-2 infection, approximately 81% of patients will develop a mild disease that, in the majority of cases, will end in symptom resolution and complete recovery, while in 33%, at least one persistent symptom will occur, constituting long COVID syndrome [2,3]. In 14% of infected patients, however, especially in those with predisposing factors, such as age > 60 years, increased body mass index (BMI), chronic comorbidities, and immunosuppression [4,5], clinical deterioration is common, leading to intensive care unit admission and death, resulting in an overall COVID-19 case-fatality rate of 2.3% [6] and a hospital case-fatality rate of 13.6% [7].

As of April 2022, 65% of the world population has received at least one dose of a COVID-19 vaccine. Despite this breakthrough, COVID-19 will constitute an enduring public health issue [8]. Vaccine hesitancy [9], breakthrough infections [10], waning immunity [11], new variants [12], and persistent transmission despite vaccination [13] represent only some of the reasons that vaccines alone cannot completely control the pandemic [8]. Improvements to other aspects of management can supplement vaccines’ actions and efficacy. These may include new approaches in preventing infection by containing transmission from asymptomatic individuals [1] or protecting exposed individuals from infection [14], but also in rapid diagnosis and outpatient oral alternatives [15]. In early 2021, EMA approved the first oral antiviral against COVID-19, i.e., nirmatrelvir/ritonavir (NMV/r) [16]. We aim to review current data pertaining to the present experience and future directions regarding the use of nirmatrelvir/ritonavir in clinical practice

## 2. Methods

Broad searches of PubMed, Scopus, and Embase between 1 January 2020 and 10 November 2022 were conducted using the following keywords: ‘nirmatrelvir/ritonavir’, ‘nirmatrelvir’, and ‘Paxlovid’. Relevant publications were identified based on the titles and abstracts. No restriction on type of paper was set. Only English language papers were included in this review and the main focus was put on clinical data. Two reviewers independently screened all title/abstracts and hand-searched references of retrieved articles. Disagreements were discussed and duplicates were removed.

## 3. Opportunities for Early Intervention: Nirmatrelvir/Ritonavir

A stereotypical viral kinetic pattern characterizes SARS-CoV-2 infection with a high peak viral load during the first several days of infection, a subsequent short rapid decay period followed by a slower clearance phase of variable duration, and, finally, a rapid elimination phase [17]. In the past, antiviral medications have demonstrated efficacy in treating various infectious diseases in the early stages of disease, reducing morbidity, mortality, and the likelihood of onward transmission [18]. Concerning COVID-19, modeling studies predicting the effects of antiviral treatments suggest better outcomes with an early approach. Assuming the viral shedding duration is a relevant surrogate of COVID-19 severity, an early test-and-treat approach is highly promising for limiting the likelihood of severe disease [17]. In order to reduce peak viral load (measured by RT-PCR and observed at day 5 post onset of symptoms) by more than two logs, drug efficacy needs to be >90% if treatment is administered after symptom onset; an efficacy of 60% could be sufficient only if treatment is initiated before symptom onset [19]. A systematic review and meta-analysis showed that the mean incubation period of COVID-19 was 5.74 days [20], further strengthening the argument for early treatment.

Therapeutic targets in the viral life cycle are based on four pillars [21]: (A) binding, i.e., preventing SARS-CoV-2 binding to ACE2 receptor (mAbs, convalescent plasma or plasma-derived therapies, and vaccines fall within this category); (B) entry/exit, i.e., preventing viral/host interaction and endosome maturation (for example, ACE2 inhibitors and human protease inhibitors); (C) proteolysis, i.e., prevention of protein formation involved in viral replication with protease inhibitors of 3CLpro or papain-like protease (PLpro), which will be our main topics of discussion herein; and (D) RNA replication, i.e., prevention of viral genome replication through RNA polymerase inhibitors.

**Figure 1:**
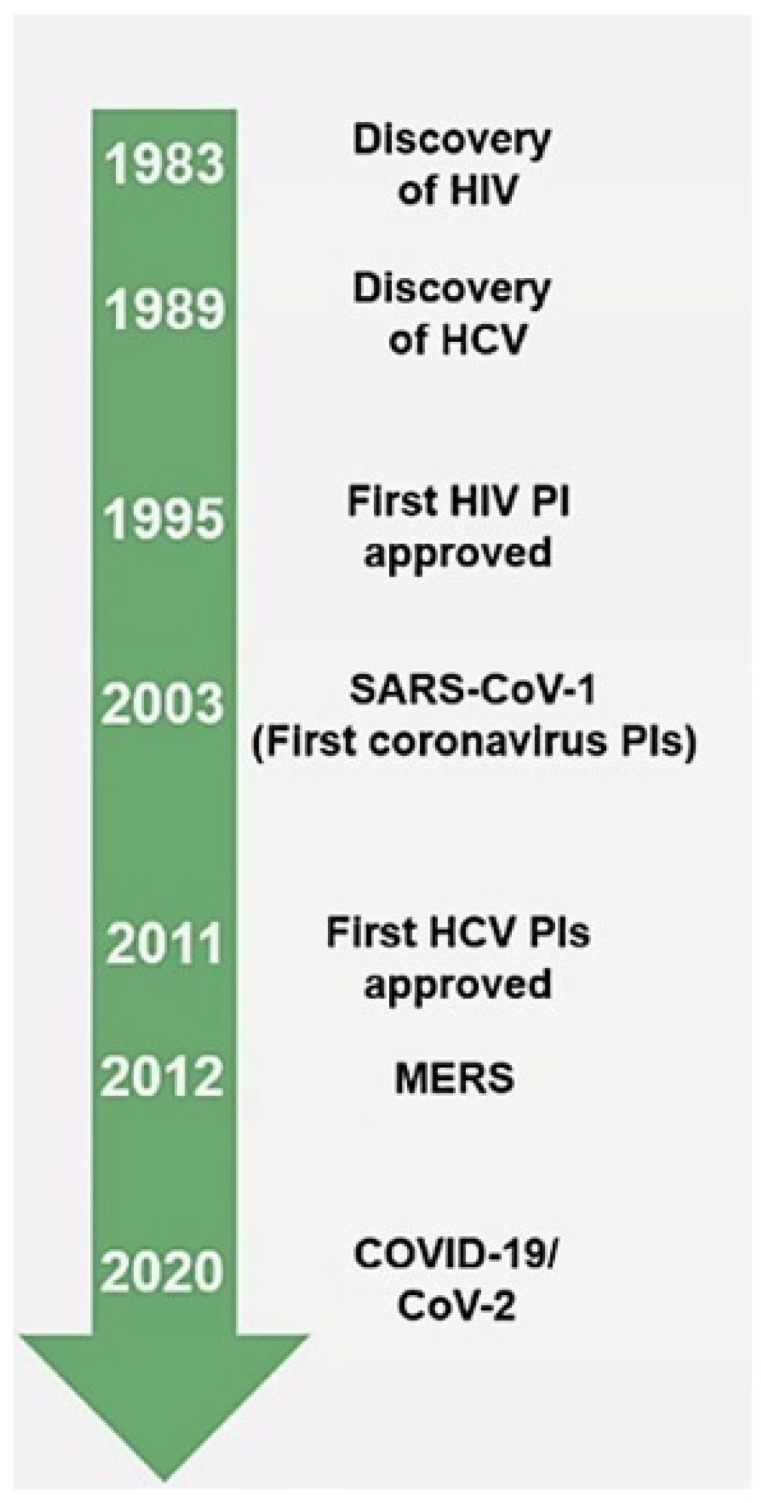
Protease inhibitors (PIs) evolution.

Protease inhibitors (PIs) are precedented to treat hepatitis C (HCV) and human immunodeficiency viruses (HIV) (Figure 1) [22] by blocking the essential processing of viral proteins for replication. 

The precedent suggests that protease inhibitors work clinically at concentrations above those shown to eradicate 90% of the virus in *in vitro* assays [23]. A correlation between viral load reduction and *in vitro* drug concentration for HCV and HIV has been noted, and viral load measurement may be used as a clinical measure of efficacy [24,25,26]. In addition, proteases are not subject to immune escape, unlike the spike protein of SARS-CoV2, and therefore protease inhibitors are predicted to be effective against variants [27].

PF-07321332, also known as nirmatrelvir, has been designed to inhibit Mpro, one of the most attractive drug targets among coronaviruses, since it is essential for viral replication and its substrate-recognition pocket is highly conserved [23,24,28]. PF-07321332 binds to the active site of Mpro and forms a reversible covalent bond with the catalytic cysteine residue, Cys145. Inhibition of Mpro renders the protein incapable of processing polyprotein precursors, thereby preventing viral replication [24,25,28]. The 3CL recognition sequence (Leu-Gln↓Ser-Ala-Gly) is not associated with any known human proteases, hence theoretically avoiding interaction with human cells. PF-07321332 demonstrated antiviral activity against all variants of concern, including Omicron and its variants [29].

Ritonavir (RTV) is an oral antiretroviral medication originally developed to treat HIV that constitutes a strong CYP3A inhibitor [30]. RTV thus enhances the bioavailability of nirmatrelvir by slowing its metabolism by CYP34A and is not expected per se to mediate any antiviral activity against SARS-CoV-2 [24]. Most common side effects are observed at high doses (600 mg BID); hence, only a low dose is used in combination with other PIs as a pharmacokinetic (PK) enhancer [30].

## 4. Administration

Patients receive the NMV/r (300 mg/100 mg) treatment course orally every 12 h for 5 days [31]. The regimen should be administered as soon as possible after the diagnosis of COVID-19 and within 5 days of symptom onset. The full 5-day treatment course should be completed, even if the patient requires hospitalization due to severe or critical COVID-19 after starting treatment with NMV/r.

Two pink 150 mg tablets of PF-07321332 and one white 100 mg tablet of ritonavir should be taken concurrently, with or without food. The tablets must be swallowed whole and not chewed, broken, or crushed, as no data are currently available. If the patient misses a dose of NMV/r within 8 h of the time it is usually taken, the patient should take it as soon as possible and resume the normal dosing schedule. If the patient misses a dose by more than 8 h, the patient should not take the missed dose and instead take the next dose at the regularly scheduled time. Double dosing to make up for a missed dose is not recommended.

No dose adjustment is needed in patients with mild renal impairment (eGFR ≥ 60 to <90 mL/min). However, the regimen should be avoided in patients with severe renal impairment (eGFR < 30 mL/min, including patients with end-stage renal disease (ESRD) under hemodialysis). In patients with moderate renal impairment (eGFR ≥ 30 to <60 mL/min), the dose of NMV/r should be reduced to 150 mg/100 mg, twice a day for 5 days, to avoid overexposure (even though no clinical data are available for this dose adjustment). Mild or moderate hepatic impairment does not require dose adjustment, but the regimen should not be used in patients with severe hepatic impairment.

## 5. A Word on Interactions

NMV/r is an inhibitor of CYP3A; hence, medicinal products that are primarily cleared by CYP3A are prone to significant increases in exposure when co-administered. In such cases, products highly metabolized by CYP3A and for which elevated plasma levels are related to severe and/or life-threatening events are contraindicated.

In addition, ritonavir has a high affinity for several CYP isoforms and may inhibit oxidation with the following ranked order: CYP3A4 > CYP2D6. As a result, it may induce glucuronidation and oxidation by CYP1A2, CYP2C8, CYP2C9, and CYP2C19, leading to enhanced biotransformation of some medicinal products associated with these pathways and resulting in decreased systemic exposure to some agents. In turn, the therapeutic effect of co-administrated agents could be decreased or shortened. 

**Figure 2:**
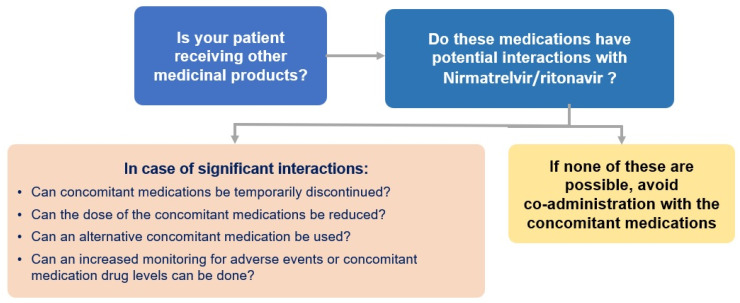
Algorithm on potential drug–drug interactions of nirmatrelvir/ritonavir.

Thus, coadministration of other CYP3A isoform substrates that could lead to potentially significant interaction should be weighed against the risks. As a conservative measure, the drug–drug interactions pertaining to ritonavir used in chronic HIV infection should apply for NMV/r. Online tools to check for interactions remain available, and regimen adjustment is recommended upon clinical judgment and on a case-by-case basis. (Figure 2) [32].

## 6. Guidelines

Currently, WHO and NIH strongly recommend NMV/r for those with a high risk of hospital admission, mostly in the context of outpatient treatment [4,33]. Other options include molnupiravir, remdesivir, and sotrovimab [32]. Therapeutic choices and characteristics are shown in Table 1. Nirmatrelvir/ritonavir represents a superior choice over other treatment options for those with non-severe illness at the highest risk. It may prevent more hospitalizations than the alternatives, has fewer harms than molnupiravir, and is easier to administer than IV options (remdesivir, monoclonal antibodies) [34].

## 7. Clinical Trial Data

NMV/r was the first per os antiviral COVID-19 medication authorized in the EU, following clinical trial results. Evaluation of Protease Inhibition for COVID-19 in High-Risk Patients (EPIC-HR) was a study of oral PF-07321332/ritonavir compared with placebo in non-hospitalized high-risk adults with COVID-19 [27]. Participants were randomized 1:1 to receive either NMV/r or placebo every 12 h for five days, while randomization was stratified by geographic region and receipt or expected receipt of mAb treatment for COVID-19. EPIC-HR assessed a composite primary endpoint of COVID-19-related hospitalization or death from any cause through day 28. Between 16 July and 9 December 2021, 2246 patients were enrolled from 343 centers worldwide. Most patients (93.8%) had not received or were not expected to receive mAb treatment for COVID-19, while 66.3% received the first dose of the trial drug or placebo within 3 days after symptom onset. A total of 61.0% of participants had ≥2 characteristics/comorbidities associated with risk of progression, including BMI ≥ 25 kg/m^2^ (80.5%), cigarette smoking (39.0%), hypertension (32.9%), >65 years of age (12.8%), and diabetes mellitus (12.2%) [27]. NMV/r resulted in significantly fewer COVID-19-related hospitalization or death events by day 28 versus placebo (*p* < 0.001). The relative risk reduction (RRR) was 89.1% and 88.9% at the interim and final analysis timepoints, respectively. NMV/r resulted in an approximately 10-fold reduction in viral load over time versus placebo when treatment was initiated within 3 days after symptom onset. The incidence of AEs that emerged during or after the treatment period (TEAEs) was similar among recipients of NMV/r (22.6%) and the placebo (23.9%). Most TEAEs in both treatment groups were mild to moderate (Grades 1–2) in severity. No patients in the NMV/r group experienced an AE resulting in death (Grade 5), while there were 13 deaths among placebo recipients.

## 8. Real-World Evidence

Real-world data on effectiveness have confirmed EPIC-HR primary results showing significant risk reduction in hospitalization and death [35,36,37,38,39,40,41,42,43,44,45,46,47,48] (Table 2).

The effect was demonstrated even among vaccinated individuals [41] in a propensity score-matched cohort of 2260 vaccinated individuals. A primary composite outcome of all-cause ER visits, hospitalization, or death in 30 days occurred in 89 (7.87%) patients in the NMV/r cohort compared to 163 (14.4%) patients in the non-NMV/r cohort consistent with 45% relative risk reduction. In terms of all-cause emergency room visits, all-cause hospitalization, and 30-day mortality, the relative risk reduction was 41, 60, and 100%, respectively [41]. The effect was also demonstrated in high-risk patients, while Omicron was the prevailing variant. In a larger retrospective cohort study of COVID-19 patients in Israel, including adults, who were at high risk for severe COVID-19, 180,351 patients were included, of whom 135,482 (75.1%) had adequate COVID-19 vaccination status [36]. NMV/r was independently associated with a significantly decreased risk for the composite outcome of severe COVID-19 or death (HR: 0.54 (95% CI: 0.39–0.75)), while adequate COVID-19 vaccination status was associated with significantly decreased risk for the composite outcome (HR: 0.20 (95% CI: 0.17–0.22)). The magnitude of NMV/r effectiveness appears to be non-related to COVID-19 vaccination status (*p*-value for interaction = 0.129), even though, it exerted one of its maximum effects in the immunosuppressed subgroup of patients [36]. Similarly, among 307 patients with systemic autoimmune rheumatic disease receiving immunomodulatory regimens, only 1.3% was recorded to suffer the composite outcome of severe COVID-19 requiring hospitalization or death [48]. 

Even though, the magnitude of risk reduction was more prominent in the EPIC-HR trial (88%) than in studies depicting real-world experience, the lower risk reduction observed in the latter could be attributed to a number of factors including heterogenicity between the studies, such as those related to the virus variant, study design, and settings. In particular, during the EPIC-HR trial, the B.1.617.2 (Delta) strain was the circulating variant (98%), while recent real-world studies were conducted at the beginning of the fifth COVID-19 wave when predominant circulation (approximately 95%) of the BA.1 variant Omicron, which is associated with lower rates of severe cases compared to Delta, was occurring [36]. As for study design differences, the EPIC-HR trial included symptomatic adults prone to more severe disease. On the contrary, real-world evidence included patients with laboratory confirmation of SARS-CoV-2 infection [36], since no information on COVID-19–related symptoms was available. In this context NMV/r administration may have been delayed in real-world cohorts, as treatment in EPIC-HR was initiated within 5 days from symptoms’ onset, while patients were included up to 5 days from SARS-CoV-2 laboratory confirmation in real life. Taking into account viral kinetics and disease pathophysiology, symptoms preceding laboratory confirmation, effectiveness is expected to be higher the earlier NMV/r is administrated. Lastly, adherence and follow up issues that have an impact on efficacy can be the least predicted and controlled for in a real-world setting.

## 9. Rebound Phenomena

Case reports have described SARS-CoV-2 viral rebound, meaning the recurrence of COVID-19 symptoms in some patients who have completed treatment with NMV/r. Case reports and results from the EPIC-HR trial even describe instances of increased SARS-CoV-2 RNA levels following the completion of the treatment course. In a cohort of 483 high-risk patients treated with NMV/r for COVID-19, two patients (0.4%) required hospitalization by day 30. Four patients (0.8%) experienced a rebound of symptoms, which were generally mild, at a median of 9 days after treatment, and all resolved without additional COVID-19-directed therapy [49]. In another cohort comprising data of 5287 patients aged >12 years (median age 61 years), out of whom 72.5% were vaccinated with three or more doses, 17.7% with two doses, and 7.8% were unvaccinated, only 6 (0.11%) hospitalizations and 2 deaths attributed to underlying disease and 39 (0.74%) emergency department encounters were seen in the defined 5–15-day period after NMV/r dispensation, representing <1% of NMV/r treated patients. Of note, all hospitalized patients had comorbidities or were >61 years of age [42]. Similarly, in a cohort of patients with systemic autoimmune rheumatic diseases receiving immunomodulatory regimens, rebound phenomena reached 8%; however no patient eventually presented severe disease, adding up in reassuring evidence [48]. 

The frequency, mechanism, and clin”cal ’mplications of these rebound events are unknown. One explanation for this rebound phenomenon is the resumption of SARS-CoV-2 viral replication following the completion of therapy, triggering a secondary immune-mediated response that manifests as a recurrence of clinical symptoms. Clinical rebound corresponds to the development of a robust antibody and T-cell immune response, arguing against a high risk of disease progression, even though the presence of infectious virus supports the need for persistent isolation [50]. It is unclear if this also represents the resumption of viral replication in persons with incompletely controlled infection due to inadequate length of therapy (5 days) or a natural biphasic pattern of viral replication. Either way, rebound phenomena are predominantly self-limiting and mild in nature, rarely requiring further management.

There are currently no data on the efficacy of administering longer courses or a second course of NMV/r. Severely immunocompromised patients can experience prolonged periods of SARS-CoV-2 replication, which may lead to rapid viral evolution, especially in the immunocompromised population [51]. There are theoretical concerns that using a single antiviral agent in these patients may produce antiviral-resistant viruses. Reportedly, amounting Mpro mutations may confer resistance to NMV/r in vitro, while resistance genes have in fact been identified in community isolates [52]. However, preliminary analysis of 731 matched day 1 and day 5 samples with available sequencing data suggests no significant associations between Mpro mutations and treatment failure [27]. Additional studies are needed to assess this risk. The role of combination antiviral therapy or a longer treatment duration in treating severely immunocompromised patients is not yet known.

## 10. Expert Option and Future Directions

NMV/r has been a game changer in the management of COVID-19. Prompt administration prevents severe disease and poor outcomes in patients with severe co-morbidities including immunocompromise. These patients will most probably first be detected by physicians primarily attending them for other chronic diseases, and not necessarily COVID-19. It is thus pivotal that, subspecialty doctors are aware and trained to recommend and prescribe early oral treatment with NMV/r according to local regulations. In this effort, potential interactions should not halter its use, since they are expected to be less than feared in number and impact. In most cases, associated regimens can be temporarily interrupted or substituted, with no or minor clinical effects. In the context of its established efficacy and safety, a ‘test-and-treat’ approach should also be discussed, even in patients with an absence of known risk factors or age constraints, following a broader cost-effectiveness analysis. In line with the above, increase of public awareness and equal opportunity to care-seeking is necessary for any intervention to achieve maximum benefit [53]. Questions, however, do remain as to repeated or prolonged rounds of administration in cases of rebound or re-infections especially, in the immunocompromised population, in line with similar experimental practices with other antivirals [54]. Lastly, even though, at the moment no resistance mutations have been identified, caution and vigilance is warranted to identify and report potential future cases. 

We are currently waiting for the results of the recently completed randomized double-blind EPIC-PEP evaluating the results of protease inhibition for COVID-19 in post-exposure prophylaxis in adult household contacts of infected individuals [55]. Work in progress includes EPID-SR [56] and EPIC IC [57] assessing the effect of NMV/r in standard risk and immunocompromised participants, respectively, while open-label EPIC-Peds will give us more insight into the effect of protease inhibition [58]. Data of NMV/r in pregnancy have also been emerging at the moment with favorable results in small cohorts, even though systematic testing has not and is unlikely to be easily performed in the clinical setting in this group of patients [47].

## 11. Limitations of This Study

This was a literature review of data regarding experience with NMV/r use in the clinical setting throughout the last two years. Even though, the literature remains limited in comparison to other pharmaceutical regimens, experience with NMV/r is rapidly expanding in the context of the existing pandemic; hence, the authors run the risk of this review being outdated by the time is published. Moreover, we included only English language papers in our search. It is possible that experiences recorded in languages other than English were missed in this review, which thus may suffer from publication and selection bias.

## 12. Conclusions

The use of protease inhibitors on an outpatient basis has the potential to alleviate a significant burden caused by COVID-19 in health systems worldwide. NMV/r’s efficacy rates and safety profile display much promise in this respect. It is crucial to raise awareness among health professionals and patients alike regarding the availability and use of NMV/r, especially among high-risk populations.

## Figures and Tables

**Table 1:** Comparison of treatment options for high-risk non-hospitalized patients.

	Absolute Risk Reduction	Relative Risk Reduction	Drug Interactions	Administration	Pregnancy	Adverse Events
**Nirmatrelvir/ritonavir**	6.3→0.8%	88%	Anticipated drug interactions of concern	Oral	Safe in pregnancy	Minor
**Remdesivir**	5.3→0.7%	87%	Few/no anticipated drug interactions	Intravenous	Safe in pregnancy	Minor
**Molnupiravir**	9.7→6.8%	30%	Few/no anticipated drug interactions	Oral	Not recommended in pregnancy	Mutagenicity
**Sotrovimab**	7→1%	85%	Few/no anticipated drug interactions	Intravenous	Safe in pregnancy	Minor

**Table 2:** Real-world data on the effectiveness of Paxlovid™.

Reference	Country of Origin	Population Characteristics	Outcomes
**Arbel et al., 2022; Oral Nirmatrelvir and Severe COVID-19 Outcomes During the Omicron Surge** [35] **https://doi.org/10.21203/rs.3.rs-1705061/v1**	Israel	Omicron B1 variant Outpatients (109,213 eligible, 3939 treated) 64% were ≥65 78% prior immunity (vaccinated or infection)	**Primary endpoint: COVID-19 hospitalization** **Secondary endpoint: Death** 68% RRR for COVID-19 hospitalization > 65 years with prior immunity and 85% without prior immunity; 79% RRR for death in people aged >65 years No impact on younger patients (confounders noted)
**Najjar-Debbiny et al., 2022; Effectiveness of Paxlovid in Reducing Severe COVID-19 and Mortality in High-Risk Patients** [36] **https://doi.org/10.1093/cid/ciac443**	Israel	Omicron B1 variant Outpatients (180,351 eligible, 4737 treated) 79% were ≥60 75% adequately vaccinated	**Composite Endpoint:** Severe COVID-19 or COVID-19 specific mortality 46% RRR for Pax, 80% RRR for vaccine Effectiveness similar in vaccinated and unvaccinated
**Wong et al., 2022; Real-world effectiveness of molnupiravir and nirmatrelvir; ritonavir among COVID-19 inpatients during Hong Kong’s Omicron BA.2 wave: an observational study** [37] **https://doi.org/10.1016/S1473-3099(22)00507-2**	Hong Kong	Omicron B2 variant Hospitalized patients not initially O_2_-dependent (1856 molnupiravir recipients, 890 Pax recipients and matched controls) 82% were ≥65 10% fully vaccinated, no prior COVID infections	**Composite Endpoint:** death, IMV, or ICU admission 43% reduction in composite endpoint for Pax Shorter length of hospital stays in survivors 40% reduction in composite endpoint for molnupiravir
**Wong et al., 2022; Real-world effectiveness of molnupiravir and nirmatrelvir; ritonavir against mortality, hospitalization, and in-hospital outcomes among community-dwelling, ambulatory COVID-19 patients during the BA.2.2 wave in Hong Kong: an observational study** [38] **https://doi.org/10.1108/2022.05.26.22275631**	Hong Kong	Omicron B2 variant Non-hospitalized patients (1072,004 eligible, 5257 received molnupiravir and 5663 Pax) 68.5% were ≥65 35% fully vaccinated	**Endpoints:** Death, COVID-19-related hospitalization, and composite of in-patient death, IMV, or ICU admission 31% reduction in hospitalization; 75% reduction in death; protection of Pax > molnupiravir
**Yip et al., 2022; Impact of the use of oral antiviral agents on the risk of hospitalization in community COVID-19 patients** [39] **https://doi.org/10.1093/cid/ciac687**	Hong Kong	Omicron B2 variant Non-hospitalized patients (83,154 controls, 5808 molnupiravir, 4921 Pax recipients) Average age was 71 years old 43% fully vaccinated	**Primary endpoint:** Hospitalization **Secondary endpoint:** Death, IMV, or ICU admission 21% reduction in hospitalization with Pax, no impact with molnupiravir No difference for secondary endpoint
**Ganatra S et al., 2022; Oral Nirmatrelvir and Ritonavir in Non-hospitalized Vaccinated Patients with COVID-19** [41] **https://doi.org/10.1093/cid/ciac673**	USA	Vaccinated Non-hospitalized (1130 patients and equally matched controls) Mean age 57.6 years, 63% female	**Primary outcome:** Composite ER visits, hospitalization, or death **Secondary endpoints:** Individual components and symptoms 45% RRR in primary composite outcome Ten deaths noted, all in the non-Pax cohort
**Dryden-Petersonet, S et al., 2022; Nirmatrelvir plus ritonavir for early COVID-19 and hospitalization in a large US health system** [40] **https://doi.org/10.1108/2022.06.14.22276393**	USA	Omicron B2 variant (lineages BA.1.1, BA.2, and BA.2.12.1) Non-hospitalized patients (24,286 controls, 6036 were prescribed Pax) Individuals aged 50 years and older 87.2% vaccinated	**Primary endpoint:** Hospitalization D14 Hospitalization in 40 (0.66%) with Pax and 232 (0.96%) control (adjusted RR: 0.55 (95% CI: 0.38 to 0.80, *p* = 0.002)) 39 deaths control vs. no deaths Pax Low baseline rate with greatest impact in unvaccinated and obese patients
**Malden DE et al., 2022; Hospitalization and Emergency Department Encounters for COVID-19 After Paxlovid Treatment—California, December 2021–May 2022** **MMWR (cdc.gov)** [42]	USA	Outpatient population (*n* = 5287) 47% were >65 y 73% vaccinated with 3 doses 56.7% Pax recipients with >1 medical condition	**Endpoints:** Hospitalization or ER COVID-19-related illness during the 5–15 days after Pax < 1% of all patients 6 patients hospitalized and 39 ER were either unvaccinated or vaccinated with 1 dose (17.8%) vs. all treated patients (9.7%), 2 deaths (multiple comorbidities and age)
**Allen S et al., 2022; Game Changer: Paxlovid Reduces Hospitalization and Saves Lives** **(epicresearch.org)** [43]	USA	Cosmos Data December 2021–August 2022 N(Pax) = 29,334 N(control) = 1,963,129	**Endpoints:** Hospital admission, death, or discharge Hospitalization: Pax 1.86% vs. control 9.6% Death: Pax 0.12% vs. control 1.23% Patients prescribed Pax ~5× less likely to be hospitalized and 10× less likely to die after COVID-19 Patients > 45 years much more likely to be prescribed Nirmatrelvir/r
**Lewnard et al., 2022; Effectiveness of nirmatrelvir-ritonavir against hospital admission: a matched cohort study in a large US healthcare system** [44] **https://doi.org/10.1101/2022.10.02.22280623**	USA	Non-hospitalized patients (4329 received Pax, 20,980 served as control) 93.3% vaccinated with 2 doses 78.1% vaccinated with 3 doses 94.2% symptomatic	**Primary endpoints:** Effectiveness against hospital admission and acute respiratory infection (ARI)-associated hospital admission over 15 and 30 days Pax dispensed within 0–5 days of symptom onset; 88.1% (95% CI: 49.0–97.5%) over 15 days and 71.9% (25.3–90.0%) over 30 days effectiveness in preventing any admission 88.3% (12.9–98.8%) over 15 days and 87.3% (18.3–98.5%) over 30 days effectiveness in preventing ARI-associated hospital admissions Pax initiation at any point of disease; 86.6% (54.9–96.3%) and 78.0% (46.2–91.4%) effectiveness in preventing all hospital admissions over 15 and 30 days, respectively 93.7% (52.5–99.4%) and 92.8% (53.9–99.1%) in preventing ARI-associated hospital admissions over 15 and 30 days, respectively Similar effectiveness regardless of total vaccine doses (≥2 doses)
**Gentile et al., 2022; Nirmatrelvir/Ritonavir and Molnupiravir in the Treatment of Mild/Moderate COVID-19: Results of a Real-Life Study** [46] **https://doi.org/10.3390/vaccines10101731**	Italy	Omicron wave 257 outpatients (56.8% received molnupiravir, 43.2% received Pax) 96.1% vaccinated	**Endpoints:** 1.6% overall hospitalization rate over 14 days 2.1% hospitalization in molnupiravir group vs. 0.9% in Pax 12.1% reported at least one ADR. No statistically significant difference in ADRs reported between groups (8.9% molnupiravir vs. 16.2% Pax, *p* = 0.075)
**Qian et al., 2022; Outcomes with and without outpatient SARS-CoV-2 treatment for patients with COVID-19 and systemic autoimmune rheumatic diseases: A retrospective cohort study** [48] **https://doi.org/10.1101/2022.10.27.22281629**	USA	Outpatients with systemic autoimmune rheumatic disease (N = 704,426 patients received treatment, 307 received Pax, 105 monoclonal Abs, 5 molnupiravir, 3 remdesivir, 6 combined treatment)	**Endpoint:** Outpatient treatment reduced the odds of severe COVID-19 by 88% 8% of those receiving oral treatment experienced COVID-19 rebound
**Loza et al., 2022; Short-term Pregnancy Outcomes After Nirmatrelvir–Ritonavir Treatment for Mild-to-Moderate Coronavirus Disease 2019 (COVID-19)** [47] **https://doi.org/10.1097/AOG.0000000000004900**	USA	Outpatient treatment of 11 pregnant women (7 received Pax)	**Endpoint:** No adverse perinatal effects were observed

Pax: Paxlovid^TM^, RR: risk ratio, RRR: relative risk ratio, IMV: invasive mechanical ventilation, ICU: intensive care unit, ER: emergency room, ARI: acute respiratory infection, ADR: adverse drug reaction, Abs: antibodies.

## Data Availability

Not applicable.
